# Association between Physical Literacy and Physical Activity: A Multilevel Analysis Study among Chinese Undergraduates

**DOI:** 10.3390/ijerph17217874

**Published:** 2020-10-27

**Authors:** Rui-Si Ma, Raymond Kim-Wai Sum, Ming-Hui Li, Yan Huang, Xue-Liang Niu

**Affiliations:** 1 School of Physical Education, Jinan University, Guangzhou 510632, China; penny@link.cuhk.edu.hk (R.-S.M.); niuxueliang53@gmail.com (X.-L.N.); 2 Department of Sports Science and Physical Education, Faculty of Education, The Chinese University of Hong Kong, Hong Kong, China; venuslmh@link.cuhk.edu.hk (M.-H.L.); Hayleyhy@link.cuhk.edu.hk (Y.H.)

**Keywords:** physical literacy, physical activity, association, relationship, Chinese undergraduates

## Abstract

Aim: To examine the association between the perceived physical literacy (PL) and physical activity (PA) levels among Chinese undergraduates. Methods: Simplified Chinese version of the Perceived Physical Literacy Instrument and the International Physical Activity Questionnaire were used to measure 536 students’ perceived PL and PA levels. Pearson’s product-moment correlation and multiple linear regression were then used to examine the relationship between the perceived PL and PA levels. Additionally, standard regression analysis was conducted to test for the effects at different demographics. Results: The correlation between perceived PL and PA level was low but significant (r = 0.350, *p* < 0.01). The multiple linear regression equation was significant (F = 25.228, *p* < 0.01, ΔR^2^ = 0.120). Metabolic equivalent values were used to predict PA levels of participants, which were −3818.582 + 272.535 (motivation) + 249.848 (confidence and physical competence) + 149.899 (interaction with the environment). The association of factors such as socio-economic status (SES) (*p* = 0.092) and grade point average (GPA) (r = 0.119, *p* = 0.022) were examined using Pearson’s product-moment correlation. Gender (*p* < 0.01) and body mass index (BMI) (*p* < 0.01) were also explored for their differences. Conclusions: Perceived PL and PA levels were significantly related. The association between PL and GPA was identical. GPA and BMI showed significant difference between each group. The study offers a path to explore the concept of PL and how it can affect the PA of Chinese undergraduates. Furthermore, on the basis of this study, more research could develop practical interventions for Chinese undergraduates to enhance their PL and engagement in a lifetime of PA.

## 1. Introduction

The concept of physical literacy (PL) rapidly gained global attention and led to a research boom [1,2]. Given its potential value for transforming society from a physical activity (PA) suppressed culture to a PA rich one [3], many countries began to promote PL in various environments, including schools, communities, and public health institutes [4]. The United Nations Educational, Scientific and Cultural Organization (UNESCO) stated that PL is a key component of physical education (PE) [5]. England Youth Sport Trust set PL as the basis of PE and school sport [6,7]. The Society of Health and Physical Educators (SHAPE) America revised the outcome of K-12 PE to cultivate PL people [8]. The World Health Organization (WHO) also stated that PL should be aligned with health when developing a national action plan on PA [9]. Although most institutes and researchers referenced the definition from Whitehead claimed: “the motivation, confidence, physical competence, knowledge and understanding to value and take responsibility for engagement in PA for life” [10], each country and organization promoted their own PL on the basis of cultural difference. Canada adopted the concept early and it flourished in communities [11]. The Aspen Institute, in the United States, drew up ability, confidence, and desire as key words for people to have PL [12]. Australia added a social dimension to the concept [11]. New Zealand, likewise, added a spiritual dimension [11]. Based on flourishing research, a growing body of work exists that examines the issue of measurement in the PL domain. Canada launched the passport for life program to assess the PL of students and teachers through active participation and living, fitness, and movement skills [13]. Physical Literacy Assessment for Youth (PLAY) was developed by Canada Sport for Life to examine PL. PLAY has six separate instruments for different groups of people [14]. The Canadian Assessment of Physical Literacy for children and youth (CAPL) was created to measure PL in four dimensions, namely, physical competence, daily behavior, motivation and confidence, and knowledge and understanding [15].

Drawing from the current research on PL assessments, many researchers are looking for extensions and applications of PL in cross-sectional fields. Cairney and colleagues introduced a conceptual model that positions PL as a health determinant and concluded that PL can be measured [16]. Jefferies and colleagues showed a link between PL and resiliency [17]. Kwan and colleagues also conducted an intervention study to examine the impact of PL on PA behaviors and fitness in university students [18]. Roetert and colleagues established the bridge between PL and PE. However, non-English speaking regions had difficulty adopting PL because of cultural differences. Raymond and colleagues first invented a perceived physical literacy instrument (PPLI) to measure the PL of students and PE teachers in Hong Kong [19,20] and tested the relationship between PL and PA in Hong Kong adolescents [21]. Then, Ma and colleagues translated PPLI into a simplified Chinese version (PPLI-SC) for testing PL among Chinese undergraduates [22].

Although Mainland China announced a policy to develop PL among students [23], the emerging concept still remains at a superficial stage. Most previous studies were limited by translated PL as sports literacy or PE literacy. Researches on the localization of the concept of PL remain slow and without a solid conclusion. Thus, more research should be conducted to improve physical activity (PA) through PL. Exploring the relationship between PL and PA will be the first step. As Whitehead stated, PL is not equal to PA, nor is it a PA substitute. The relationship between PL and PA is one of facilitation. PL does not have to be manifested in PA, people, including those who are not able to perform any PA, can demonstrate and benefit from PL [11]. PL is the predecessor of PA, which is developed through PL [24,25]. Studies exist that focus on the relationship between PL and PA for different age groups in other countries and regions [21,26,27]. Studies found that children’s PL was associated with their PA the authors suggested future research to test the causality of the association [27]. Other study proved that among Hong Kong adolescents, instead of being forced to participate in PA, individuals will take an active part in PA if they understand PL. Concurrently, individuals will enhance their PL by joining in PA, then forming a beneficial cycle [21]. Moreover, moderate-to-vigorous PA was proposed to be the combination of PL and PA in school-age children [26]. For university students, Kwan and colleagues found the PL-based intervention program was effective in helping university students attenuate PA decline in the first year, though the underlying mechanisms remain unclear [18]. However, Mainland China has yet to conduct such research. This study thus aimed to test the causality of the association between PL and PA in Mainland China. PL was collected by using PPLI-SC [22]. Different from the original Cantonese version, PPLI-SC re-collected 18 items to test the reliability and validity among Chinese undergraduates and then developed a unique instrument that fits the local culture. For PA, PA level was used to present the behavior and exercise intensities of undergraduates [28,29]. The subject was the last stage of the education process from primary school, high school, and college [30]. Young people at this stage should take PA seriously because it is a significant part of being healthy [31]. Given that undergraduates are essentially on campus daily, university plays a vital role in developing and maintaining continued PA participation. Understanding the relationship between PA and PL can help improve the school’s PE courses, which enhances students’ health. In addition, Patricia and colleagues proposed that individual factors such as gender, educational factor, and socioeconomic status (SES) might impact the PL [24]. There was little research testing the relationship between PL and PA in different demographic groups. This study will be the first to explore PL in relation to demographic groups among Chinese undergraduates.

## 2. Method

### 2.1. Design

Cross-sectional study was utilized in this research. Participants’ perceived PL and PA level were calculated by PPLI-SC and a simplified Chinese version of the long version of the International Physical Activity Questionnaire (IPAQ-SC). Pearson’s product-moment correlation and multiple linear regression were then used to examine the relationship between the perceived PL and PA level. The demographic information (gender, grade, GPA, and so on) of participants were also collected for further correlation analysis. The ethical approval was obtained from the Medical Ethics Committee of the First Affiliated Hospital of Jinan University.

### 2.2. Participants

Jinan University was chosen as the location for the research. The Ministry of Education of the People’s Republic of China expressed that all Mainland China universities must complete 144 h of PE courses within four years [32]. In this way, each university student has some common characteristics in PA behavior. Participants were randomly invited to join this research. A total of 536 undergraduate students aged between 18 and 21 participated in the study. Each participant was required to finish a questionnaire, which includes demographic information, PPLI-SC, and the long IPAQ-SC form. Study details were fully explained to the participants beforehand. Before completing the questionnaire, all participants were asked to provide informed consent that he or she volunteered to participate in the research.

The research assistant was responsible for distributing the questionnaire to each participant and for answering the participant’s questions during this period. Each participant was allowed to ask questions throughout the questionnaire. In addition, they were free to refuse answering the questionnaire and could withdraw at any time. To encourage participation, some simple gifts, such as pens and bookmarks, were given to them once they finished the survey. After the participants finished the questionnaire, all questionnaires were collected by the research assistant.

### 2.3. Measures

Participants’ perceived PL was assessed by PPLI-SC, which was translated from PPLI. It is an eight-item instrument used to measure the perceived PL of Chinese undergraduates. It includes three dimensions, which are (1) motivation, (2) confidence and physical competence, and (3) interaction with the environment. To be specific, (1) physically literate individuals will maintain positive attitudes toward PA throughout their lives. (2) Physically literate people are able to move with confidence and poise in a variety of challenging situations. (3) Physically literate people can interact with the environment in the context of everyday living [31]. All of these dimensions were described as kernel attributes of Whitehead’s concept of PL. The participants responded on a 5-point Likert scale (1 = strongly disagree to 5 = strongly agree). The outcome of PL is to sum the number up (the total score ranges from 8 to 40). PPLI-SC was proved to be a reliable and valid questionnaire to measure perceived PL of Chinese undergraduates through exploratory factor analysis (factor loadings ranged from 0.68 to 0.93) and confirmatory factor analysis (factor loadings ranged from 0.60 to 0.92) [22].

Participants’ PA levels were measured by the long version of IPAQ-SC, which was translated from the long version of IPAQ. It involves five environment domains: work-related (unrelated in this study), transportation, housework, leisure time, and sedentary. These domains were used to record participants’ moderate and vigorous PA performed for at least 10 uninterrupted minutes in the previous 7 days (5 school days and 2 weekend days). The data were collected in minutes per week for each of the PA intensities. Then, minutes were calculated for metabolic equivalent (MET) values by using the MET-minutes computation formulation [33,34]. Qu NN and Li KJ translated IPAQ into a simplified Chinese version and tested its reliability and validity. Reliability was used for the test-retest method (*p* < 0.05) and validity was used for Spearman’s rank correlation established by Caltrac accelerometer (*p* < 0.05, r = 0.50). The results showed that the long version of IPAQ-SC had intraclass correlation coefficients above 0.7 for PA [35].

The demographic information of the participants, such as gender and BMI, was also collected in the questionnaire. GPA was a standard way to measure academic achievement; Jinan University used a 5-point GPA model. Students with a GPA of less than 3 were allocated a normal score, GPA between 3 and 4 meant a good score and GPA higher than 4 meant an excellent score [36]. The SES factor was used to evaluate students’ socioeconomic status. According to the National Bureau of Statistics of China, SES was divided into three levels: RMB 0–4000 equals a low income, RMB 4000–20,000 equals medium, and higher than RMB 20,000 means high SES [37].

### 2.4. Data Analysis

IBM SPSS 25 was used for data analysis. The correlation between perceived PL and PA level was derived from Pearson’s product-moment correlation. Before the regression analysis, the research team examined the normality, linearity, and multicollinearity of the data to ensure that no violations existed during the analysis. Multiple regression analysis was then used to detect the association between perceived PL and PA levels. In addition, multiple regression analysis was conducted separately for males and females. Measures, such as demographic information, GPA, and SES were also considered in the Pearson’s product-moment correlation and standard regression analysis in computing the correlation with PL. The difference of each gender’s PL was explored through student’s *t*-test. Each type of BMI was valued in PL by using analysis of variance (ANOVA) and z-scores were calculated in the correlations to test the influence on the relationship between PA and PL.

## 3. Results

Before testing the relationship between PA and PL, demographic characteristics of participants were analyzed. A total of 622 undergraduates were recruited and 536 of them completed the questionnaires (female = 403; male = 133). The participants’ ages ranged from 18 to 21 years old. The average age of participants was 19.40 years old and standard deviation was 0.83, which were all consistent with the research subjects.

Pearson’s product-moment correlation was used to test the correlation between each dimension of PA level and three attributes of perceived PL (Table 1). The average of total perceived PL of participants was 30.28 (17–40, standard deviation ±4.20) and the average of three domains, (1) motivation, (2) confidence and physical competence, and (3) interaction with the environment were 11.92 (5–15, ±0.25), 11.09 (3–15, ±0.29), and 7.27 (2–10, ±0.23), respectively. The daily average time the participants spent on moderate PA and vigorous PA were 7.34 (2–60, ±9.06) minutes and 4.94 (1–34.42, ±6.9) minutes, respectively. The daily average transportation time of students was 10.57 (1–58.14, ±8.73) minutes, which included 1.71 (0–27.4, ±0.12) minutes riding, 0.14 (0–17.14, ±0.04) minutes cycling, and 8.5 (3–25.7, ±0.32) minutes walking. Daily average housework and leisure time were 4.29 (0–60, ±6.74) minutes and 13.52 (0–62.85, ±12.5) minutes.

The correlation (r) between participants’ perceived PL and PA levels was 0.35, which was significant at the 0.01 level (2-tailed). Although the correlations are not high, three dimensions of PPLI-SC, namely, intensities of PA (walking, moderate PA, and vigorous PA) and three domains of IPAQ-SC (transportation, housework, and leisure time) showed a high significance (r = 0.088–0.336, *p* < 0.01). No significant correlation existed between sedentary domain of IPAQ-SC and any other attribute.

Multiple linear regression was used to predict the PA levels of Chinese undergraduates on the basis of the three dimensions of perceived PL (Table 2). The analysis showed a significant regression equation (F = 25.228, *p* < 0.01, ΔR^2^ = 0.120) and so did the dimensions of perceived PL. The predicted PA level of participants was −3818.582 + 272.535 (motivation) + 249.848 (confidence and physical competence) + 149.899 (interaction with the environment) MET value. The standardized coefficients of (1) motivation, (2) confidence and physical competence, and (3) interaction with the environment were 0.176, 0.184, and 0.087, respectively. Except for interaction with the environment, the probability values of the other two dimensions were all less than 0.05, which showed a positive relationship between perceived PL and PA level. The participants’ MET values would increase by 272.535, 249.848, and 149.899 for each dimension score of perceived PL. The results showed that two dimensions were significant predictors of PA level.

Multiple linear regression was then used to examine males and females separately (Table 3 and Table 4). The results showed a significant regression equation both in males (F = 9.219, *p* < 0.01, ΔR^2^ = 0.157) and females (F = 15.243, *p* < 0.001, ΔR^2^ = 0.098). For PL dimensions in males, only motivation showed significant probability value (*p* = 0.048). For females, the probability values of motivation and physical competence were both less than 0.05.

SES (*p* = 0.092) and GPA (r = 0.119, *p* = 0.022) factors also examined the association by using Pearson’s product-moment correlation. Except for physical competence (r = 0.039, *p* = 0.461), motivation (r = 0.116, *p* = 0.022) and interaction with environment (r = 0.131, *p* = 0.012) both showed significant correlation with GPA. Table 5 shows the results of the standard regression analysis to predict PL using GPA (F = 5.294). For each point increase in GPA, the perceived PL score will is enhanced by 0.857. Comparison of the different PL in gender (t = 2.174, *p* < 0.01) was explored by student’s *t*-test. Perceived PL of males (31.02, ±4.97) were higher than females (29.98, ±3.88). BMI (F = 7.381, *p* = 0.001) was classified into three types and analyzed giving their different PL through ANOVA. BMI was classified into three types, namely, low, medium, and high. The medium group showed significant differences with low (*p* = 0.001) and high (*p* = 0.015) groups. For further analysis, z-scores were calculated to explore the different influences of each group in the relationship between PL and PA (Table 6). Except for the high GPA group (r = 0.025, z = 0.025), all other groups showed significant positive correlation between PL and PA (r = 0.338–0.529, r = 0.352–0.589).

## 4. Discussion

Although studies that focus on the objectively assessed PL in the world are increasing [14,15,16,17,18,19,20], studies focusing on groups in Mainland China are lagging in this area. This study thus focused on exploring the relationship between perceived PL and PA level among Chinese undergraduates and investigating demographic factors related to PL. As assumed, the results showed significantly positive relationships between PA and PL. Males and females both showed significance separately, but differences between them were found. In addition, GPA showed significant positive relationship with PL while SES did not. Meanwhile, students with normal BMI showed significant difference both with high and low BMI.

### 4.1. Relationship between PL and PA of Undergraduates in Mainland China

Nearly half of the research claimed that PL is the core of PA, and individuals would not participant in PA if they had low PL [24]. According to such general research, PL will show a strong correlation with PA. However, the result of this study showed that they have a significant but relatively low correlation. Although such result was inconsistent with most literature, it showed high consistency with previous exploration in Hong Kong adolescents [21]. Such low positive correlation reflected that when the concept of PL has not been integrated into PE courses, students may not have much sense of PL even though some of them have high levels of PA.

This study used three dimensions of PL as predictive factors to test the PA level among Chinese undergraduates by using multiple regression analysis. By enhancing the dimension of motivation, students could find more interest in participating in PA and thus will likely maintain this positive attitude toward PA throughout their lives. From developing the dimension of confidence and physical competence, students will move their bodies with elegance and balance, which will enhance their confidence to join more PA. Moreover, by increasing the chance of environmental interaction, students could build more self-confidence, which will encourage them to attend PA again. Given that PE courses are the main way for Chinese undergraduates to become involved in PA, the concept of PL should be assigned into PE courses to improve students’ PL. With in-depth understanding of the concept of PL, students will value and likely engage in PA [29,30].

### 4.2. Relationship between Perceived PL and Various Factors of PA Level

Exploring the relationship between perceived PL and PA level will help to find effective ways to improve the PA levels of Chinese undergraduates [22]. On the basis of PPLI-SC and IPAQ-SC, this study conducted an analysis on the association between perceived PL and various relevant factors of PA level. According to the health recommendations of the WHO, adults should participate in PA, including moderate and vigorous PA, for at least 30 min per day [9]. Most undergraduates failed to meet the recommendation. This may cause the low correlation between PL and PA. The result also showed that most factors of PA (transportation, walking, housework, and so on) affect PL and its three dimensions.

As aforementioned, a certain amount of PA is required to achieve the health standard, but the length of PE courses in Mainland China universities was not sufficient to meet this requirement [23]. Moreover, most students participated in little, moderate, or vigorous PA [38]. Therefore, this results in a relatively low correlation between perceived PL and PA level (r = 0.350). PE courses are the main way for most students to participate in PA. However, PE courses are not the only factor related to PL, PA, such as transportation and housework will also increase the core attributes of PL. Such PAs in daily life are also vital to understanding the intrinsic value of PL. Among these various activities, PA during leisure time has the highest correlation with PL. In recreational activities, the attribute of free choice of PA can awaken individuals’ interests, and this attribute also has the highest performance (r = 0.336). As Whitehead stated, self-directed selection of PA can effectively improve PL [11]. More than that, by freely choosing PA, individuals can actively participate in games confidently and therefore enhance physical competence. Thus, other core attributes in PL, confidence and physical competence, can effectively be improved [31]. This dimension also showed a relatively high correlation in the relationship between PL and PA in leisure time (r = 0.291). Regarding intensity of PA, both moderate and vigorous PA were related to PL [27]. Vigorous activity (r = 0.284) is more significantly relevant than moderate (r = 0.106) because the former can better stimulate physical ability and enhance individual confidence. Especially considering their age group, younger adults such as university students, will find interest in vigorous activities. No correlation exists between sedentary activities and PL. Nowadays, sitting time, such as reading, homework, and playing video games, almost occupies most of students’ daily lives, this is the main reason why Chinese undergraduates had low PA levels. From 1991 to 2006, the PA level of Chinese adults fell by 32% [39]. Although Government released policies and set a high requirement for PE courses [40,41], participation in PA outside of school was almost nonexistent because Chinese adolescents were under academic achievement pressure [42]. In such situations, simplifying the use of PE courses to enhance students’ PA and PL is not enough. Encouraging students to engage in PA outside of class, especially during leisure time, is urgent and necessary to improve their PL and PA.

### 4.3. Relationship between Perceived PL and Various Individual Factors

Gender, physical status, academic achievement, and SES of students are factors that must be considered in the relationship between PL and PA [24,28,43].

Among Chinese undergraduates, male students and female students showed some differences in the relationship between PL and PA. Although they both showed significance in PL and PA, males only have one predictor (motivation) while females have two (motivation and confidence and physical competence). This illustrated the role of the key factor of motivation in improving males PA and PL. Meanwhile, female students need both motivation and confidence to be more active in PA. This is partly because PA engagement for males is not affected by factors such as physical condition. Compared with males, females are more likely to be influenced by interest, self-confidence, and physical condition. More influential factors lead to the result that female students are more likely to give up PA when they lack encouragement in any of the related dimensions. Globally, males were more active than females, thus more women suffered with physical inactivity than men in nearly every country [44]. Without compulsory participation in PE courses, females are inclined to choose not to exercise, thereby hindering the progress of PL. The result showed that males’ perceptions of PL was significantly different than females’. According to the finding that males and females prefer different PA intensities [45], PE courses must be redesigned by focusing on all students’ (especially females) PA preferences, confidence in themselves, and interaction with one another [31]. For this reason, PA habits developed on campus will make students take responsibility of lifelong physical exercise.

Body management is considered one of the key factors that can affect individuals’ PL [31,33,46]. Entire body movement, such as balancing and jumping require good physical status. Individuals with good health and fitness will complete such activities easier than obese and emaciated people. The results show that students with normal body size have higher PL and it is also significantly different from the two other groups. Namely, they are likely to engage in various PAs whereas the other groups are not. In 2019, China Youth Daily reported that China University Media United launched a questionnaire to investigate the willingness of Chinese undergraduates to lose weight. The results showed that 72.73% of them were not satisfied with their body mass and 77.78% of them planned to lose weight. However, nearly 90% of them failed halfway [47]. Interestingly, high and low BMI groups showed nearly the same PL score. Therefore, the concept of PL should be integrated into students’ daily lives to awaken their motivation to participate in PA and to value it as a lifestyle, thereby continuously engaging in it.

Academic achievements positively relate to PA level [43,48,49]. Consistent with these findings, this study showed the same results in relationship between GPA and PL (*p* = 0.022). For each 1 point score increase in GPA, a corresponding 0.857 enhancement in PL exists. Thus, the higher transcript a student has, the more he or she would be willing to participate in PA. However, when viewed in combination with the z-scores, there is a ceiling on the GPA. When students reached GPAs higher than 4, their PL would not be increased. For dimensions of PL, motivation (r = 0.116, *p* = 0.022) and interaction with environment (r = 0.131, *p* = 0.012) showed significant positive correlation with GPA. Thus, considering both enthusiasm for participating in PA and interaction with the environment will increase students’ academic performances, PE courses can start from these two points to build curriculum content that is conducive to all-round development of Chinese undergraduates. Different from other two dimensions, physical competence showed no significant correlation with GPA. This also raised a question for traditional PE courses, which in the past were focused on improving students’ physical competence rather than motivation. The SES of family is positively related to the PA of students [50,51]. Students from higher social economic families are likely to join each PA and to maintain the lifetime pattern because their families can support them better in materials and education [21]. However, results from this study showed that in Mainland China, families’ SES does not relate to PL. Tomporowski and colleagues stated that families of different SESs tend to participate in different types of PA [52]. Based on the same sport types that PE courses offered and most Chinese undergraduates spent most of their time in university, concluding that the PE course systems of Chinese universities showed a good example in excluding the impact of SES of families to some point is reasonable. However, it will be good for giving more choices to let low SES students have the opportunity to participate in sports that high SES students will choose. In the long run, whether making PE courses compulsory, like China has, will increase involvement in PA might be the direction of future research. Based on this study, future research could continue investigation into how to increase PA of Chinese undergraduates through enhancing PL.

## 5. Limitation

This study contains certain limitations. First, because the participants of this research were randomly collected, female undergraduates accounted for a large portion, which may cause outcome deviations. Second, subjects of this research all are Chinese undergraduates (from 18–21 years old), further study could continue to explore the association in other age groups. Third, PL and PA in this study were all collected from subject data (questionnaires of PPLI and IPAQ), further research could use objective measures for accuracy. Fourth, this study did not collect information about student majors, which may show a possible correlation in PA and PL. Further study could continue to explore and discuss this field.

## 6. Conclusions

This study aims to examine the relationship between perceived PL and PA levels among Chinese undergraduates through a cross-sectional study given the lack of research focused on the topic. Additionally, the research investigates the relationship between PL and related factors such as gender, BMI, GPA, and SES. The results showed that attributes of perceived PL and PA level were associated with most of domains of PA and factors of individual demographic. Hence, the concept of PL should not only be incorporated into PE courses, but also into their daily lives as a lifestyle. The research focuses on the subjects of university students, which is the last stage of the education process following on from primary school, high school, and college. We highlight the importance of association between PA and PL, which showed a way to explore the concept of PL and how it can affect the PA of Chinese undergraduates. Furthermore, based on this study, more research could develop practical interventions on Chinese undergraduates to enhance their PL to influence them to engage in a lifetime of PA for wellness and longevity.

## Figures and Tables

**Table 1:** Pearson’s product-moment correlation of 3 attributes of perceived physical literacy (PL) and physical activity (PA) domains (*n* = 536).

	PA Level	Moderate PA	Vigorous PA	Transportation	Housework	Leisure Time	Sedentary
Total PL	**0.350 ^a^**	0.106 ^b^	**0.284 ^a^**	0.218 ^a^	0.128 ^a^	**0.336 ^a^**	0.018
Motivation	**0.289 ^a^**	0.096 ^b^	0.274 ^a^	0.164 ^a^	0.116 ^a^	**0.283 ^a^**	0.072
Confidence and physical competence	**0.296 ^a^**	0.092 ^b^	0.234 ^a^	0.185 ^a^	0.093 ^b^	**0.291 ^a^**	−0.018
Interaction with the environment	0.220 ^a^	0.055	0.142 ^a^	0.153 ^a^	0.088 ^b^	0.198 ^a^	−0.010

Correlation higher than 2.80 was bolded. ^a^ Correlation is significant at the 0.01 level (2-tailed). ^b^ Correlation is significant at the 0.05 level (2-tailed).

**Table 2:** Results of multiple linear regression analysis predicting PA level using perceived PL and its dimensions (*n* = 536).

Variable	B ^a^	SEB ^b^	Ρ ^c^	P ^d^
Constant	−3818.582	837.967	-	0.000
Motivation	272.535	72.159	0.176	0.000
Confidence and physical competence	249.848	63.635	0.184	0.000
Interaction with the environment	149.899	77.192	0.087	0.053
R = 0.353				
R^2^ = 0.125				
ΔR^2^ = 0.120				

^a^ Unstandardized coefficient. ^b^ Standard error of unstandardized coefficient. ^c^ Standardized coefficient. ^d^ Probability value.

**Table 3:** Results of multiple linear regression analysis predicting PA level using perceived PL and its dimensions in males (*n* = 133).

Variable	B ^a^	SEB ^b^	Ρ ^c^	P ^d^
Constant	−4930.002	1608.180	-	0.003
Motivation	296.795	148.927	0.195	0.048
Confidence and physical competence	242.393	132.688	0.181	0.070
Interaction with the environment	253.295	163.879	0.143	0.053
R = 0.420				
R^2^ = 0.177				
ΔR^2^ = 0.157				

^a^ Unstandardized coefficient. ^b^ Standard error of unstandardized coefficient. ^c^ Standardized coefficient. ^d^ Probability value.

**Table 4:** Results of multiple linear regression analysis predicting PA level using perceived PL and its dimensions in females (*n* = 403).

Variable	B ^a^	SEB ^b^	Ρ ^c^	P ^d^
Constant	−3363.262	1012.700	-	0.001
Motivation	249.064	83.775	0.159	0.003
Confidence and physical competence	266.391	75.453	0.191	0.000
Interaction with the environment	109.543	88.944	0.065	0.219
R = 0.324				
R^2^ = 0.105				
ΔR^2^ = 0.098				

^a^ Unstandardized coefficient. ^b^ Standard error of unstandardized coefficient. ^c^ Standardized coefficient. ^d^ Probability value.

**Table 5:** Results of standard regression analysis predicting PL using GPA (*n* = 536).

Variable	B ^a^	SEB ^b^	Ρ ^c^	P ^d^
Constant	27.691	1.169	-	0.000
GPA	0.857	0.373	0.119	0.022
R = 0.119				
R^2^ = 0.014				
ΔR^2^ = 0.012				

^a^ Unstandardized coefficient. ^b^ Standard error of unstandardized coefficient. ^c^ Standardized coefficient. ^d^ Probability value.

**Table 6:** Pearson’s product-moment correlation and z-score of perceived PL and PA level in each factor (*n* = 536).

	Gender		BMI			GPA			SES		
	Male	Female	Low	Medium	High	Low	Medium	High	Low	Medium	High
N	133	403	153	319	64	144	344	48	80	306	150
r	0.467 ^a^	0.346 ^a^	0.338 ^a^	0.370 ^a^	0.366 ^a^	0.393 ^a^	0.338 ^a^	0.025	0.342 ^a^	0.374 ^a^	0.529 ^a^
z	0.506	0.361	0.352	0.388	0.384	0.415	0.352	0.025	0.356	0.393	0.589

^a^ Correlation is significant at the 0.01 level (2-tailed).
